# Redefining the Tea Green Leafhopper: *Empoasca onukii* Matsuda (Hemiptera: Cicadellidae) as a Vital Asset in Premium Tea Production

**DOI:** 10.3390/life15010133

**Published:** 2025-01-20

**Authors:** Unisa Conteh Kanu, Zhaohong Wang, Chenshi Qiu, Qiaojun Wen, Xueyan Li, Dongliang Qiu, Yinwei Gan, Runqian Mao

**Affiliations:** 1 Guangdong Key Laboratory of Animal Conservation and Resource Utilization, Guangdong Public Laboratory of Wild Animal Conservation and Utilization, Institute of Zoology, Guangdong Academy of Sciences, Guangzhou 510260, China; kanuunisa2@gmail.com (U.C.K.); wangzh@giz.gd.cn (Z.W.); 18239287859@163.com (X.L.); 2 Zijin Agricultural Comprehensive Service Center, Yongtai Street, Zijin, Heyuan 517400, China; chaye0762@163.com (C.Q.); 5220330053@fafu.edu.cn (Q.W.); 3 College of Horticulture, Fujian Agriculture and Forestry University, Fuzhou 350002, China

**Keywords:** significant enhancer, flavor profiles, premium teas, secondary metabolites, niche markets

## Abstract

This review explores the evolving role of the tea green leafhopper, *Empoasca onukii*, in the tea industry, transitioning from a recognized pest to a significant enhancer of tea quality. Recent research highlights how its feeding behavior stimulates the production of desirable secondary metabolites, thereby improving the flavor profiles and market value of premium teas, particularly varieties like Taiwan’s “Oriental Beauty”. As consumer demand for unique and artisanal teas rises, the economic benefits associated with *E. onukii* are becoming increasingly evident, prompting farmers to adopt sustainable agricultural practices that often involve reduced pesticide use. Furthermore, the dynamic interplay between climatic factors, *E. onukii* population dynamics, and tea cultivation practices necessitates integrated pest management strategies that balance the beneficial and detrimental impacts of this leafhopper. Understanding these complexities not only fosters sustainable production methods but also opens niche markets, benefiting local economies and promoting both economic viability and environmental sustainability in the tea industry.

## 1. Introduction

The tea green leafhopper, *E. onukii*, a member of the Hemiptera order and family Cicadellidae [1,2,3], was once considered a nuisance due to its harmful effects on tea plants. However, recent research has reevaluated its potential economic benefits to the tea industry, highlighting its ability to enhance the quality and market value of certain tea cultivars, particularly those with unique flavor profiles that are increasingly sought after by consumers [4,5,6,7]. One of the most noteworthy economic advantages of this small green leafhopper is its capacity to improve the flavor profile of tea leaves [2,8,9]. By feeding on the sap of tea plants, *E. onukii* triggers a range of protective biochemical reactions that increase the synthesis of secondary metabolites such as flavonoids, terpenoids, and volatile organic compounds (VOCs) [4,6]. These compounds add distinct and highly desirable flavor notes to tea. For instance, recent studies conducted by the authors of [7,8,10,11,12] have shown that *E. onukii* enhances the production of compounds like linalool, geraniol, and hotrienol, which are associated with the fruity and floral aromas characteristic of premium teas. This biological response is particularly significant in the production of “Oriental Beauty” tea, a renowned Taiwanese oolong tea celebrated for its honey-like sweetness and floral fragrance.

As consumer demand for teas with unique, premium flavor profiles increases, the market value of leafhopper-affected teas has risen dramatically [13]. These products are successfully marketed as high-end, artisanal goods that command much higher prices due to the complexity and uniqueness imparted by *E. onukii*’s activity. For instance, “Oriental Beauty” tea, often referred to as Bai Hao Oolong, can cost several hundred dollars per kilogram [14]. This trend is not limited to Taiwanese teas; similar practices are being adopted in other tea-producing regions like China and Japan, where the flavors induced by leafhoppers are gaining popularity.

This development allows tea producers to distinguish their offerings from mass-produced alternatives, creating a niche market that appeals to luxury buyers and connoisseurs willing to pay a premium for uniqueness and quality. Additionally, *E. onukii* offers economic advantages for sustainable agriculture. As demand for teas influenced by this insect species rises, some farmers have reduced or eliminated pesticide use traditionally aimed at controlling it, aligning with global movements promoting environmentally friendly farming. Regions that tea plantations adopting pesticide-free practices to support leafhopper activity have seen decreased production costs related to chemical inputs and a reduced environmental footprint [12,15,16].

These sustainable practices enhance the marketability of tea products, particularly among environmentally conscious consumers, providing an additional layer of economic advantage. The renewed interest in leafhopper-affected teas has also revitalized artisanal tea production techniques, preserving traditional methods in regions like Taiwan that associate these products with heritage and craftsmanship [17,18]. This cultural and historical significance, alongside distinctive flavor profiles, adds significant economic value and attracts both domestic and international customers. The demand for such premium, leafhopper-influenced teas has positively impacted local economies, particularly in rural tea-growing areas, as it necessitates specialized labor for planting, harvesting, and processing [19,20].

Furthermore, the growing popularity of these teas has further fueled the mass rearing of *E. onukii*. By enhancing flavor profiles, developing specialized markets, and promoting sustainable farming practices, the mass rearing of *E. onukii* provides notable economic benefits to the tea industry. For example, the premium flavor profiles of these teas primarily arise from the biochemical reactions triggered by the leafhopper’s feeding activity. Tea farmers can consistently produce high-quality products that command premium prices by cultivating and managing *E. onukii* populations [7,16,21].

This approach fosters local economies and tea-related tourism while spurring research and development, making the insect an invaluable asset to the sector. However, while challenging, mass rearing of *E. onukii* is feasible and, if executed properly, can significantly enhance the economic sustainability of the tea industry [22]. Through strategic host plant selection, meticulously controlled environmental conditions, and effective management of leafhopper populations, tea growers can harness the distinctive benefits of *E. onukii* to cultivate premium, commercially viable teas that cater to discerning consumers. As this technique evolves, it is poised to significantly contribute to the profitable and sustainable production of tea in the future. Whether managing leafhopper populations in tea gardens or engaging in large-scale breeding for the production of “hopper tea”, a systematic and comprehensive understanding of the tea leafhopper is essential.

## 2. What Is *E. onukii*?

**Figure 1:**
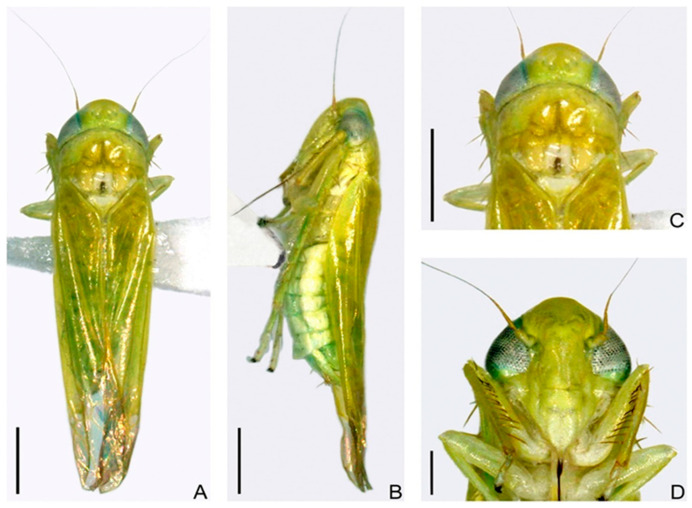
An image of the *E. onukii* (Hemiptera: Cicadellidae) Matsuda [1]. (**A**) Male adult, dorsal view. (**B**) Female adult, left lateral view. (**C**) Head and thorax, dorsal view. (**D**) Face. Scale bars: (**A**–**C**) = 0.5 mm; (**D**) = 0.2 mm.

There are several distinct species of small green leafhoppers in tea gardens, and because of their striking physical similarities, it can be challenging to distinguish between populations without looking at the male reproductive organs [18,23]. As a result, based only on morphological evaluations, numerous regional surveys and investigations have distinguished between distinct species of little green leafhoppers that are predominant. Studies of leafhoppers in various ecoregions have mentioned several names; therefore, the dominant species in tea gardens has been controversially identified over the last decade. The main leafhopper species damaging tea gardens in Japan is acknowledged to be the little green leafhopper *E. onukii* [24]. In the meantime, scientists in Taiwan, China, identified *Jacobiasca formosana* (Paoli) as the dominant species in tea plantations [1,3,25,26]. These results underline the need for additional research into the species classification and phylogenetic links of the little green leafhoppers that are identified by different regions of the world as the primary pests in tea gardens. It had a varied diet and was not very damaging. However, the invasion of tea gardens by this pest became a major problem when tea cultivation spread widely in China’s mountainous and semi-mountainous regions. The precise name and taxonomic classification of the dominant species of tea green leafhopper in Chinese tea gardens, however, remain a matter of controversy. The first study by the authors of [1,25,27] revealed that the false-eyed leafhopper, *Empoasca vitis* Göthe, is probably the prevalent species in China’s tea gardens. This study includes the morphological identification of small green leafhopper specimens from tea gardens in 11 provinces. However, the taxonomic classification of the dominating species of tea green leafhopper was not resolved by further investigation. In response, the authors of [28,29] confirmed the findings in [27,30] by performing a thorough examination of the reproductive organ structure of tiny green leafhoppers from tea estates in several Chinese regions in 2000. Since then, this finding has been widely accepted and is still cited. Furthermore, the authors of [2,31] used RAPD molecular techniques to examine small green leafhoppers from different tea-producing regions. They discovered that these leafhoppers’ genetic makeup is largely consistent across different regions, supporting the false-eyed leafhopper’s identification. Using mitochondrial 16S rRNA and COI gene sequences, the authors of [30] further identified tea green leafhoppers as false-eyed leafhoppers by determining that there were no significant differences across 12 geographical populations. Similarly, using mitochondrial COI gene sequences, the authors of [30] examined the evolutionary relationships between tea leafhoppers and their close relatives. They found that the 12 populations of tea leafhoppers had genetic distances ranging from 0.5% to 1.2%, which is significantly less than the 2% threshold commonly used to identify insect species. The genetic distances, on the other hand, varied from 14.8% to 24.5% between tea leafhoppers and their more distant relatives, which include the grape small green leafhopper, grape two-star leafhopper, and peach speckled leafhopper. Then, using different mitochondrial gene sequences, the authors of [25,30] examined tiny green leafhoppers from tea gardens in Taiwan, Japan, and the Chinese mainland. The leafhoppers from these three regions have been given separate scientific names in the literature, but their research has shown that there are only slight genetic variations between the populations, indicating that they are conspecific. Then, utilizing external reproductive organ inspection, the authors of [1] analyzed male small green leafhoppers from tea gardens in Taiwan, Japan, and the Chinese mainland. It was suggested that the leafhoppers from these areas are members of the same species, which is the little green leafhopper known as *E. onukii*, which is found in Japanese tea gardens (Figure 1).

Furthermore, the authors of [1,29], who dissected the reproductive organs of male small green leafhoppers in the Shaanxi and Fujian tea regions, independently verified this conclusion. It was confirmed by additional extensive surveys and sample analyses conducted by the authors of [28,32] that *E. onukii* is the predominant species in most tea-growing locations. As a result, researchers have begun to identify the tiny green leafhoppers in tea gardens in Taiwan, Japan, and mainland China as *E. onukii*. The term “tea green leafhopper” is still used in this study to describe *E. onukii*.

## 3. Taxonomic, Phylogenetic Groups, and Distribution

**Figure 2:**
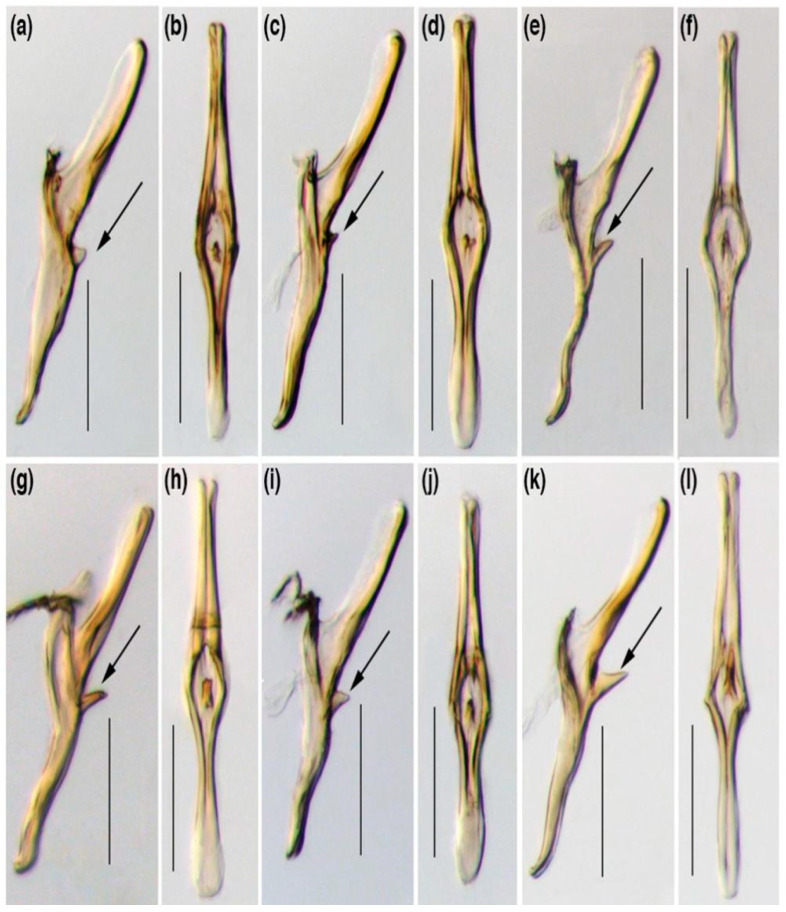
Morphological variations of aedeagus of *E. onukii* from different tea production regions of Yunnan Province. (**a**) Chuxiong (CX) population, aedeagus; (**b**) Chuxiong (CX) population; (**c**) Pu′er (PE) population; (**d**) Pu′er (PE) population; (**e**) Mojiang (MJ) population; (**f**) Mojiang (MJ) population; (**g**) Lincang (LX) population; (**h**) Lincang (LX) population; (**i**) Jingdong (JD) population; (**j**) Jingdong (JD) population; (**k**) Menghai (MH) population; (**l**) Menghai (MH) population. (**a**,**c**,**e**,**g**,**i**,**k**) aedeagus, left lateral view; (**b**,**d**,**f**,**h**–**j**) aedeagus, ventral view. Scale bars: (**a**–**l**) = 0.1 mm. Arrows display variations in the shape of the dorsal membranous flanges and the length of ventro-basal spiny protuberances of the aedeagal shaft [33].

Although previous studies conducted by the authors of [1,3,28,32] described both Taiwan, Japan, and mainland China as a single species, recent comparative morphological studies indicated significant intraspecific variation among populations, with individuals from various southern Chinese populations differing in the shape of their membranous flanges and the length of spiny protrusions on the shaft of the male aedeagus [1,32,33] (Figure 2).

**Figure 3:**
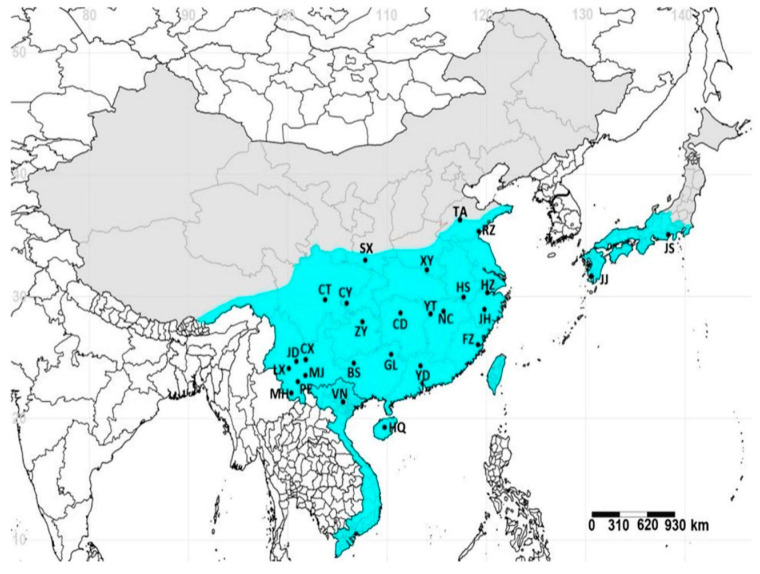
Geographic distribution and populations of *E. onukii* [33].

This insect species is currently widespread across Japan, Vietnam, and China, which are subject to different ecological conditions [1,25]. Given these previous morphological findings, the extensive distribution of the species, and the varying environmental conditions of tea-growing regions, we proposed that these morphological differences reflect genetic variations among geographic populations (Figure 3).

Previous research on the population structure and genetic differentiation of the tea green leafhopper has primarily relied on mitochondrial gene sequences or a limited number of microsatellite markers [1,25,26,30,34]. Results from microsatellite analysis indicated some geographical differentiation between populations, especially between two populations in Yunnan and other Chinese populations, but provided limited evidence of consistent population genetic structure. This inconsistency may be influenced by the inherent properties of the markers (such as fluctuations in allele frequency and recombination rates), as well as the quality of genetic data and sampling methods.

**Figure 4:**
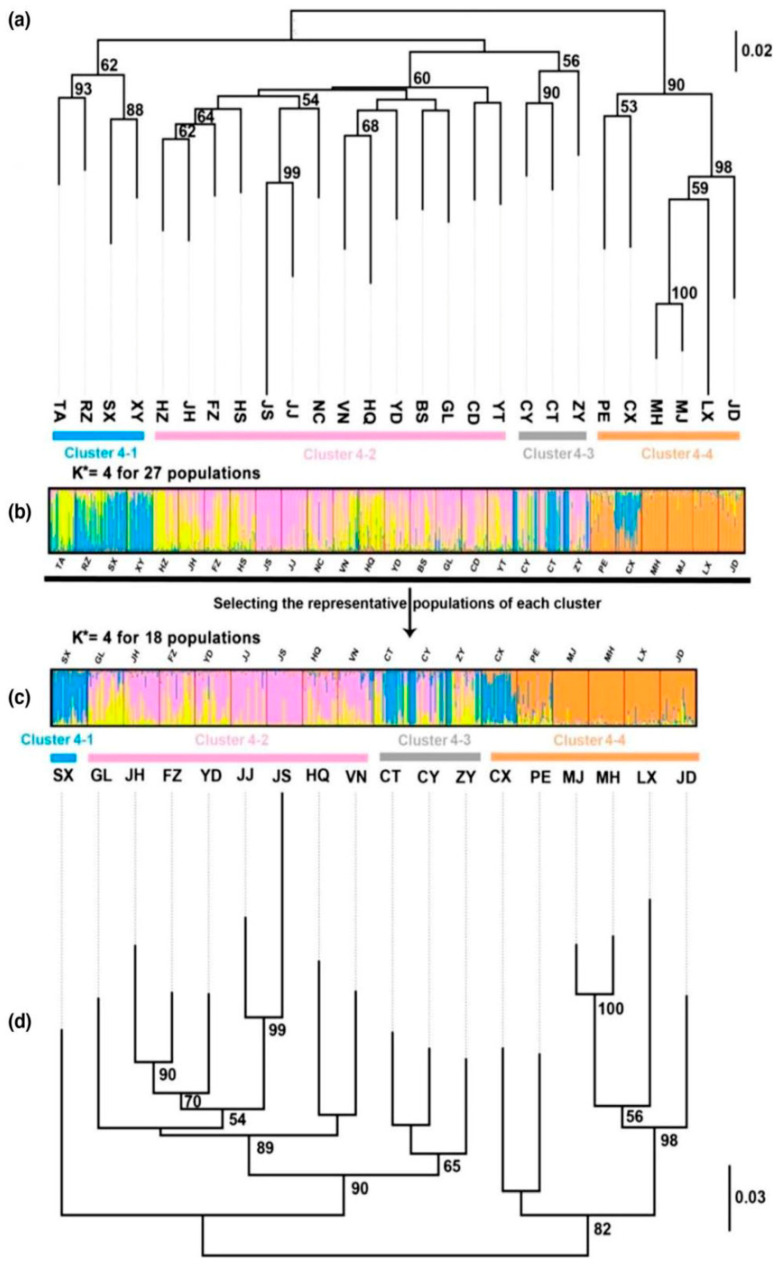
Bayesian clustering results and neighbor-joining (NJ) tree of *E. onukii* populations based on 18 microsatellite markers. Numbers on nodes represent bootstrap support values (values < 50% not shown). The colors indicate the major clusters inferred by Bayesian clustering analysis when K = 4, except in Clusters 4–3 where mixed genetic components are indicated by gray. Asterisks represent the optimal K = 4. (**a**,**d**): NJ tree for 27 populations and 18 populations; (**b**,**c**): Bayesian clustering results for 27 populations and 18 populations [33].

The authors of [33] genotyped samples from 27 sites that represented 18 geographical populations of *E. onukii* throughout East Asia using 1633 single nucleotide polymorphisms (SNPs) and 18 microsatellite markers (SSRs). Significant genetic differentiation and organization were found, with Yunnan populations showing the highest levels in comparison to other populations. SSR data revealed that the Japanese populations of Kagoshima (JJ) and Shizuoka (JS) clustered with particular Chinese populations, such as Jinhua (JH) and Yingde (YD), as well as a Vietnamese population (Vinh Phuc VN), despite SNP analysis suggesting that these populations were distinct from Chinese populations (Figure 4).

Conversely, SSRs allowed the CT, CY, ZY, and Shaanxi (SX) populations to be distinguished from one another while still grouping together based on SNPs. The study discovered a number of variables that may have an impact on the insect’s quick spread throughout Asia, including physical obstacles, human transportation of the host plant (*Camellia sinensis*), and regional climate adaptations. The study shows that high-throughput SNP genotyping can successfully and broadly reveal minute genetic features in r-strategist insects. The authors of [2] also distinguished four unique genetic groups from several sample sites, including Yunnan, Southern-Central China, and Eastern China. The wide geographic range of *E. onukii* and the diverse ecological condition of tea cultivation account for the significant genetic diversity seen in populations from these ecoregions. For management techniques to be effective, *E. onukii* must be accurately identified and classified. Erroneous identification may result in inadequate management strategies and a misapprehension of the biology and ecology of the insect. Understanding the taxonomy and systematics of *E. onukii* contributes to broader efforts in biodiversity conservation and the study of insect diversity in agricultural systems.

## 4. Biology of *E. onukii*

**Figure 5:**
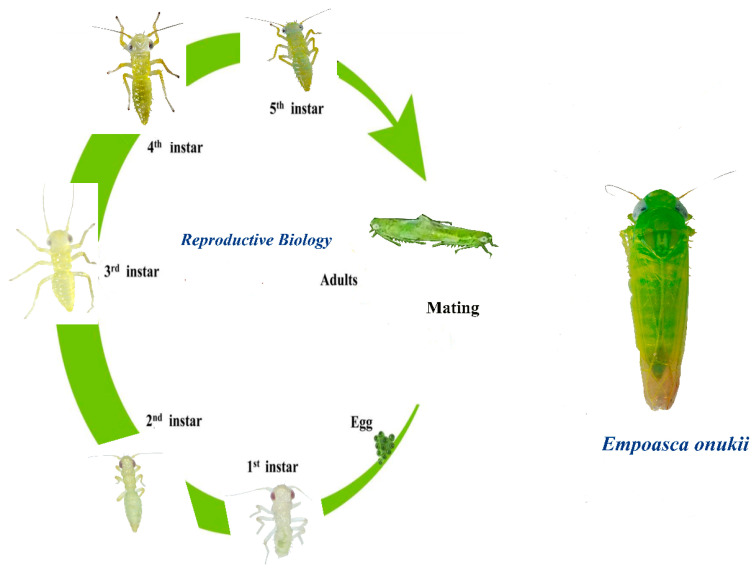
Seven stages of *E. onukii* reproductive biology. Egg produced through five nymphal instar stages, with final differentiation into adult males and females.

*E. onukii* undergoes hemimetabolous development, characterized by the absence of a pupal stage typical of holometabolous insects. The life cycle of the tea leafhopper, *E. onukii*, is a fascinating journey that unfolds through three primary stages: egg, nymph, and adult [35,36]. It all begins when female leafhoppers lay their eggs on the tender leaves of tea plants. These eggs are protected by a waxy coating that shields them from predators and environmental stresses [35]. Once they hatch, they emerge into tiny nymphs, which involves several molts before they reach adulthood (Figure 5).

Upon hatching, these nymphs typically go through five distinct instar stages. During this time, they not only grow in size but also develop wing pads, setting the stage for their transition to adult life [37]. The duration of each nymphal stage is influenced by external factors such as temperature and humidity (Table 1). Under optimal conditions—around 25 °C with high humidity—they may develop within 14 to 18 days. Once they have completed their nymphal stages, they molt into adults, marking a significant milestone in their life cycle [36,37].

After maturing, nymphs develop into adults, which can reproduce a few days after reaching sexual maturity. Adult *E. onukii* typically live for one to two months and can produce multiple generations in a single growing season, with studies indicating up to 10 to 12 generations per year in warmer climates [1,3]. According to the authors of [42], adult *E. onukii* engage in mating activities that are primarily influenced by vibrational signals. This reproductive behavior is particularly intriguing due to its reliance on vibrational communication for mating. After reaching adulthood, males establish territories and produce acoustic signals that attract females. Females respond to these signals, and mating typically occurs in the early morning or late evening when environmental conditions are favorable for vibration transmission. The authors of [42] reported that mating occurs only once for females during their lifetime, while males may mate multiple times. The effective communication signals for courtship involve the use of pulses and ongoing duets, making the vibrational signaling system critical for successful reproduction. The developmental outcomes are highly contingent upon environmental factors. Temperature, humidity, and host plant characteristics significantly impact their development rates and reproductive success. For instance, higher temperatures can accelerate development but may also lead to decreased survival rates if temperatures exceed optimal levels [28,43].

Similarly, the availability of suitable host plants influences nymph survival and growth. *E. onukii* prefers to feed on young, tender leaves, which provide appropriate nutrients for growth and development [28,36,44]. Any changes in the availability of suitable host plants can, therefore, affect the population dynamics and life cycle of *E. onukii*. Understanding the developmental biology of *E. onukii* is pivotal for implementing effective management strategies, particularly given its role as a significant pest of tea crops. Continuous research into its life cycle, reproductive strategies, and environmental interactions will aid in developing robust control measures that minimize the reliance on chemical pesticides, thereby enhancing environmental sustainability.

## 5. Environmental Influence to *E. onukii*

The populations of *E. onukii* are significantly shaped by climatic conditions, thriving in warm and humid environments. Many tea-growing regions create ideal microclimates for the species, resulting in population peaks during the warmer months [41,43]. Factors such as rainfall and temperature, relative humidity, and the availability of young tea leaves play crucial roles in their life cycle and population dynamics [45]. Research indicates that optimal temperatures (between 25 °C and 31 °C) can significantly enhance reproduction rates, underscoring the importance of climate in managing *E. onukii* populations. According to [39,46], the average development times for eggs and nymphs are 8 and 14.1 days at constant 25 °C and 5.8 and 8.4 days at 31 °C. It takes the just emerged adults 4–5 days to reach sexual maturity and begin mating.

### 5.1. Rainfall and Heatwaves

Rainfall and heatwaves are key environmental factors that significantly affect the population dynamics of *E. onukii* in tea gardens, impacting their reproduction, survival, and effects on tea quality. Rainfall can have both positive and negative impacts on *E. onukii*. Heavy rains can decrease populations by physically displacing nymphs and adults from tea plants. The authors of [47] noted that intense rainfall events can lower survival rates by disrupting feeding and increasing vulnerability to predation and environmental stress. Rainwater can also dilute the nutrient content on leaf surfaces, making them less attractive for feeding.

While heavy rainfall can reduce populations, moderate rainfall increases humidity, which enhances the reproductive success of *E. onukii*. The authors of [45,48] found that moderate rainfall, which maintains high humidity levels, is positively associated with increased egg-laying and hatching rates. The moisture from rain aids in egg hydration and nymphal development, supporting rapid population growth once conditions stabilize. Additionally, rainfall promotes the growth of lush tea foliage, which provides a more suitable habitat and abundant food source for *E. onukii*. The authors of [39] observed that rain-induced foliage growth leads to higher leafhopper densities due to the increased availability of tender leaves, preferred by *E. onukii* for feeding and oviposition.

On the other hand, heatwaves, with their extended periods of extreme temperatures, can put significant stress on *E. onukii*, leading to higher death rates. The authors of [49] found that when temperatures rise above 35 °C, both nymphs and adults struggle to survive due to dehydration and physiological stress. These conditions not only affect their survival but also disrupt the normal development of eggs and nymphs, which ultimately reduces their ability to reproduce successfully. Moreover, extreme heat changes how *E. onukii* feed; in these hot conditions, leafhoppers tend to eat less to avoid overheating. This shift in behavior can slow their growth and limit their population expansion. The authors of [50] showed that during heatwaves, *E. onukii* are less active during the hottest parts of the day, leading to a diminished impact on tea plants compared to when temperatures are more moderate.

Extreme heat can also disrupt the reproductive cycles of *E. onukii*. The authors of [50,51] found that heatwaves reduce the fecundity of adult females, resulting in fewer eggs laid during these periods, leading to a temporary reduction in population growth. Additionally, heatwaves affect tea plant physiology, which in turn influences *E. onukii* populations. High temperatures can cause tea plants to exhibit stress responses such as wilting or reduced sap flow, making them less suitable for feeding. The authors of [47,52] observed that during heatwaves, reduced plant sap availability diminishes leaf nutritional quality, leading to lower feeding efficiency and subsequent population declines.

Rainfall and heatwaves can have mixed impacts on tea quality through their effects on *E. onukii* populations. Moderate rainfall can boost leafhopper activity and improve tea flavor profiles by increasing secondary metabolite production. In contrast, heatwaves can reduce the benefits of leafhopper activity due to lower populations and reduced feeding.

The variability introduced by rainfall and heatwaves complicates the management of *E. onukii* in tea gardens. The authors of [53] emphasized the need for adaptive pest management strategies that consider these weather extremes to balance the leafhopper’s beneficial and harmful effects on tea production.

### 5.2. Humid Conditions

Humid conditions significantly impact the population dynamics of *E. onukii* in tea gardens, as documented in various studies. The tea green leafhopper thrives in warm, moist environments common to tea-growing regions, with high humidity enhancing its reproductive rates. For instance, the authors of [39] observed that relative humidity above 70% boosts egg-laying and hatching rates, accelerating population growth. The authors of [40] reported that the fecundity of *E. onukii* peaks at 80–90% humidity, underscoring the role of moisture in the survival and expansion of these populations. Additionally, the authors of [19] found that high humidity, along with optimal temperatures, reduces the developmental period from egg to adult, allowing more generations per year. The authors of [5,50] also noted that humid conditions speed up the nymphal stages, leading to earlier reproductive maturity and increased reproductive cycles within a single growing season. The authors of [51] highlighted that increased feeding activity occurs under moist conditions due to better leaf turgidity, which facilitates sap feeding but also intensifies plant damage, including hopperburn symptoms like leaf curling and yellowing.

Moreover, *E. onukii* disperses more rapidly in humid environments, broadening infestations and damage across tea gardens [54]. For farmers treating *E. onukii* as a pest, managing these populations under humid conditions is challenging due to their rapid growth and spread. For example, humidity diminishes the efficacy of chemical controls, as certain insecticides degrade faster in moist conditions, necessitating more frequent applications [9,55,56]. Integrated Pest Management (IPM) strategies, including biological controls and cultural practices, may need to be adjusted to counteract the rapid population increases driven by humidity [57,58,59].

On the economic front, the influence of humid conditions on *E. onukii* populations can be beneficial, particularly for high-value tea production. While traditionally seen as a pest, *E. onukii* feeding under humid conditions stimulates the production of secondary metabolites and volatile organic compounds (VOCs), which enhance tea quality and market value. The authors of [6,60] found that higher humidity levels correlate with increased concentrations of these beneficial compounds, resulting in teas with superior taste and aroma. A prominent example is Taiwan’s “Oriental Beauty” tea, where the presence of *E. onukii* contributes to its distinctive sweetness and high market value [61]. This shift towards recognizing the positive impacts of *E. onukii* under humid conditions has led some tea farmers to adopt sustainable farming practices, such as reducing pesticide use. According to the authors of [34,62], embracing organic or pesticide-free cultivation reduces input costs and appeals to health-conscious consumers, thereby boosting profitability.

These practices align with the favorable conditions for *E. onukii* populations, making humid environments ideal for producing high-value teas. This trend supports premium pricing and thus increases revenue for tea farmers, especially those in naturally humid regions. The authors of [18,63] highlighted the growing consumer demand for specialty teas influenced by *E. onukii*, driven by their unique flavors and artisanal qualities. This demand creates niche markets, enhancing economic returns and supporting local economies, particularly for small-scale farmers who market their products as premium teas [18]. Furthermore, cultivating *E. onukii*-affected teas under humid conditions can bolster broader economic activities, including tea tourism. The authors of [13] reported that such tea gardens attract tourists interested in unique production methods and tasting experiences, generating additional revenue streams for local communities.

### 5.3. Illuminance Conditions

Light intensity significantly influences the population dynamics of *E. onukii*, which in turn impacts the economic outcomes for tea cultivation. Research shows that light conditions affect the behavior, reproduction, and overall activity of *E. onukii*, thereby influencing tea quality and market value. Studies suggest that light intensity directly impacts the reproductive and feeding behaviors of *E. onukii*. The authors of [38] found that under optimal light conditions (moderate illuminance), the leafhopper’s reproductive rates increase, enhancing population growth. Higher light levels improve visibility and foraging efficiency, allowing *E. onukii* to more effectively feed on tea plant sap. This feeding stimulates the production of secondary metabolites, which are key to the unique taste and aroma profiles of high-value teas [10].

The feeding activity of *E. onukii* under favorable illuminance conditions has been linked to improved tea quality. Illuminance influences plant physiology, and in combination with *E. onukii* feeding, can lead to higher concentrations of beneficial compounds in tea leaves. The authors of [64] reported that light conditions conducive to leafhopper activity resulted in teas with enhanced flavor profiles, particularly in premium teas such as “Oriental Beauty” from Taiwan. These quality improvements translate into higher market prices, providing economic benefits to tea farmers. This condition supports the production of specialty teas, which can be marketed as premium products. According to the authors of [44,65], teas associated with *onukii* under optimal light conditions command premium prices due to their distinct sensory attributes. This market differentiation allows producers to tap into niche markets, catering to consumers who seek unique, high-quality tea experiences. The economic impact is particularly beneficial for small-scale farmers who can leverage these niche markets to enhance their income.

Additionally, optimal illuminance conditions that support healthy *E. onukii* populations also align with sustainable agricultural practices. The authors of [43,56] found that under certain light conditions, the use of pesticides can be minimized as farmers focus on maintaining the beneficial impacts of *E. onukii* on tea quality. Reducing chemical inputs not only lowers production costs but also appeals to environmentally conscious consumers, further boosting profitability.

The economic benefits of illuminance-driven *E. onukii* populations extend beyond individual farmers to local economies. The authors of [11] highlighted that tea gardens producing high-value teas under optimal light conditions often become attractions for tea tourism. Tourists are drawn to these gardens to learn about the unique production processes and taste the distinctive teas, generating additional income streams through tea sales, tours, and related activities. This integration of tea tourism provides broader economic benefits, creating jobs and supporting rural communities [4,17].

### 5.4. Cultivation Management

Cultivation management practices have a significant influence on the population dynamics of *E. onukii* in tea plantations, impacting their abundance, reproductive rates, and overall impact on tea plants. Various studies highlight how different cultivation techniques can either exacerbate or mitigate *E. onukii* infestations [48,66].

Traditional reliance on chemical pesticides to control *E. onukii* has led to the development of resistance among populations. The authors of [19] observed that frequent pesticide applications not only failed to suppress *E. onukii* effectively but also contributed to resistance, leading to higher population levels over time. Additionally, the overuse of pesticides disrupts natural predator populations, which otherwise help keep *E. onukii* numbers in check, thereby unintentionally promoting leafhopper outbreaks [45,51].

A shift towards organic and sustainable cultivation methods has been shown to influence *E. onukii* populations positively by fostering a balanced ecosystem. The authors of [5,49,67] noted that organic farming practices, including the use of natural predators like spiders and predatory insects, reduce *E. onukii* populations more effectively than conventional pesticide methods. These practices help maintain ecological balance and reduce the overall pest pressure on tea plantations.

Pruning techniques and shade management also play a role in controlling *E. onukii* populations. Pruning can directly reduce the number of feeding sites and disrupt the life cycle of *E. onukii*. The authors of [50,68] found that frequent pruning lowers leafhopper populations by removing their preferred young shoots, thus reducing available food sources and habitats for egg-laying. Similarly, managing shade levels can influence *E. onukii* populations, as these leafhoppers prefer well-lit environments. The authors of [15] demonstrated that increasing shade cover in tea plantations reduces the incidence of *E. onukii*, as lower light intensity is less favorable for their development and activity.

Fertilization practices impact the nutritional quality of tea leaves, which in turn affects *E. onukii* populations. Excessive nitrogen fertilization has been linked to higher populations of this insect pest due to the increased nutritional content of leaves, making them more attractive for feeding. The authors of [35] reported that reducing nitrogen input in tea cultivation resulted in decreased *E. onukii* densities, suggesting that careful management of fertilization can help control pest populations without compromising plant health.

Intercropping and diversification strategies can reduce *E. onukii* populations by disrupting their habitat and food preferences. Intercropping tea with non-host plants has been shown to create a less favorable environment for leafhoppers, thereby reducing their numbers. According to the authors of [47,69], intercropping tea with leguminous plants not only improved soil fertility but also lowered *E. onukii* populations by reducing the attractiveness of the habitat for leafhoppers.

Irrigation practices also influence *E. onukii* populations. Proper water management can mitigate the effects of drought stress on tea plants, which can otherwise make them more susceptible to *E. onukii* infestations. The authors of [70] found that maintaining optimal soil moisture levels reduced the population growth of *E. onukii* by improving plant vigor, thereby making the plants less appealing for leafhopper feeding and reproduction.

Implementing Integrated Pest Management (IPM) strategies, which combine cultural, biological, and chemical controls has been effective in managing *E. onukii* populations. The authors of [67] highlighted the success of IPM approaches, such as using pheromone traps, biological control agents, and selective pruning, which together create a multi-faceted strategy to keep *E. onukii* populations under control without relying solely on chemical interventions.

## 6. Host Plants’ Preference of *E. onukii*

The host plant preferences of *E. onukii* are central to understanding its population dynamics, spread, and the damage it causes. While *E. onukii* primarily targets tea plants (*Camellia sinensis*), its preference for specific plant varieties, plant parts, and environmental conditions shapes its feeding behavior, reproductive success, and management strategies [71]. Tea plants (*Camellia sinensis*) show different levels of susceptibility to *E. onukii*. This variation is influenced by several factors, including leaf texture, biochemical composition, and overall plant vigor. According to the authors of [36,68], tea cultivars with softer, thinner, and more tender leaves tend to be more prone to infestation because these leaves are easier for the *E. onukii* to pierce and extract sap from making them ideal for feeding and laying eggs. In contrast, cultivars with thicker, tougher leaves are less preferred by the pest due to the greater effort required for feeding and the physical barriers they present to the insect’s mouthparts.

The authors of [52,72] reported that cultivars with high concentrations of free amino acids, especially theanine, are more appealing to leafhoppers due to the enhanced nutritional quality of the leaves. On the other hand, cultivars with higher amounts of polyphenols, tannins, and other defensive substances tend to be less susceptible, as these compounds deter feeding and egg-laying [11,60,73]. Fast-growing, vigorous cultivars that produce a large number of young shoots and tender leaves are generally more attractive to *E. onukii*. The authors of [74,75] observed that such cultivars provide a steady supply of fresh, succulent leaves, which can sustain larger leafhopper populations. Conversely, slower-growing or less vigorous cultivars, which produce fewer tender leaves, are less likely to support significant leafhopper populations. Several studies have assessed how various tea cultivars respond to *E. onukii* attacks, revealing a range of vulnerabilities influenced by genetic, morphological, and chemical characteristics (e.g., [6,7]).

In [43], significant differences were found in the honeydew output produced by female leafhoppers on various tea cultivars. Notably, cultivar 10 produced less honeydew than the other four, while cultivars 2, 6, 7, and Fuding Da Bai had similar production levels. They also observed structural differences in leaf tissue, with cultivar 10 having a comparable leaf area to cultivars 2 and Fuding Da Bai but smaller than 6 and 7. Although its trichomes are shorter, cultivar No. 10 has a higher density of trichomes than the other varieties, potentially affecting leafhopper interactions.

Furthermore, the authors of [44], investigated how the population of *E. onukii*, varied among twelve different tea plant varieties.

The results reveal that four varieties consistently supported higher populations of these insects, while four others exhibited lower and more variable populations. The authors of [6,57] investigated the responses of two tea cultivars to leafhopper feeding. They discovered that a susceptible cultivar displayed a greater number of differentially expressed genes (DEGs) compared to a resistant cultivar after 6 and 24 h of feeding. Notably, the resistant cultivar activated its defense mechanisms earlier, primarily focusing on terpenoid metabolism and jasmonic acid production, whereas the susceptible cultivar responded more slowly. Understanding these differences aids tea growers and researchers in developing effective integrated pest management (IPM) strategies to minimize damage while maintaining high-quality tea production.

### Management Considerations and Implications

Gaining insight into the host plant preferences of *E. onukii* is crucial for understanding its growth and development in tea gardens. Identifying which specific tea cultivars appeal to *E. onukii* is key to formulating effective pest management strategies and refining cultivation methods. Extensive research has been conducted on the oviposition preferences of *E. onukii* related to various tea cultivars. Each cultivar possesses unique characteristics—such as leaf toughness, nitrogen levels, and the presence of secondary metabolites—that can significantly influence its vulnerability to this pest [36]. Collectively, these traits determine how attractive different tea cultivars are to *E. onukii* for mating and laying eggs, which in turn impacts the insect’s growth rates and population dynamics.

Research has shown that some tea cultivars are particularly conducive to the growth and reproduction of *E. onukii*. For example, investigations by the authors of [28,76] found that cultivars such as Fuding White Tea, Fuyun No. 6, and Maoxie exhibited the highest egg densities of *E. onukii*, suggesting a marked preference for these plants when it comes to oviposition. The study indicated that these cultivars possess traits that likely enhance their attractiveness to *E. onukii*, potentially due to factors like softer leaf texture or greater nutritional content.

Furthermore, additional studies have highlighted the varying levels of resistance and susceptibility among different tea cultivars. For instance, the authors of [50,77] noted that certain traditional cultivars showed lower infestations of *E. onukii* attributed to their tougher leaves and lower nitrogen content. Changes in chemical composition, such as elevated levels of polyphenols, have also been associated with reduced pest infestations, underscoring the significance of plant defense mechanisms in combating this pest [44].

For farmers viewing this insect as a threat to their tea plants and aiming to boost yield, understanding which cultivars serve as suitable hosts for *E. onukii* allows farmers to make informed choices regarding cultivar selection that balances yield and quality with pest resistance. Moreover, cultivating less susceptible tea varieties could mitigate the impact of *E. onukii* and contribute to sustainable pest management practices.

In conclusion, identifying specific tea cultivars that promote the growth and development of *E. onukii* is crucial for farmers looking to optimize tea production amid pest challenges. By leveraging insights from recent research—such as that by the authors of [39]—tea producers can devise targeted strategies to boost crop resilience, mitigate pest-related damage, and enhance the sustainability of tea cultivation. Implementing cultural practices like adjusting planting densities and pruning frequencies can effectively manage *E. onukii* populations by limiting the availability of young, tender leaves. Additionally, biological control methods, such as introducing or conserving natural predators like spiders, lady beetles, and parasitic wasps, can further help regulate *E. onukii* populations.

The economic implications of cultivating susceptible green tea cultivars for the mass rearing of *E. onukii* touch on various aspects, including pest management, crop research, market dynamics, and ecosystem sustainability. A comprehensive understanding of these factors offers valuable insights that can lead to more resilient farming practices, increased profitability, and improved product quality. To fully realize the benefits of this approach, ongoing collaboration among entomologists, agronomists, and economists is essential to foster both economic viability and environmental sustainability in the tea industry.

## 7. Feeding Behavior of *E. onukii*

The feeding patterns of the tea leafhopper, *E. onukii*, are influenced by various plant characteristics and environmental factors. Typically, *E. onukii* prefers younger, softer leaves, as they are easier to penetrate and are nutrient rich. Seasonal changes play a significant role in shaping the insect’s feeding behavior, with peak activity generally observed during the warmer months. During this time, the insect’s metabolism and reproductive rates soar. Studies, such as those by the authors of [10,35], have demonstrated a strong correlation between temperature and humidity levels with the populations and feeding damage caused by *E. onukii*. Specifically, higher temperatures combined with moderate humidity foster increased feeding activity and population growth.

This surge in feeding activity also prompts the tea plants to undergo various physiological changes. For instance, plants may develop secondary metabolites that can alter the flavor profile of the leaves or serve as defensive mechanisms. Some tea cultivars have even been selectively bred to enhance leaf toughness or boost the concentration of chemicals that deter leafhoppers. Recent work by the authors of [78,79] has unveiled a fascinating defense mechanism in certain tea cultivars, including *Camellia sinensis* var. *assamica*. When faced with the threat of leafhopper feeding, these resilient plants spring into action, releasing elevated levels of phenolic compounds. This remarkable response not only hinders the leafhoppers’ ability to feed but also disrupts their reproduction, showcasing nature’s intricate strategies for survival. By harnessing the power of these secondary metabolites, these tea cultivars are not just defending themselves; they are actively shaping their own ecological fate in the face of herbivory. This discovery highlights the dynamic interplay between plants and pests, offering valuable insights for tea growers aiming to enhance crop resilience and quality [6,80]. However, beyond these climatic effects, the resistance exhibited by various tea genotypes against *E. onukii* is an important factor to consider. Gaining insights into how different tea cultivars respond to *E. onukii* can lead to more effective management practices. For example, research conducted by the authors of [46,48] has identified certain tea genotypes that show stronger resilience to *E. onukii* infestations, highlighting the significance of genetic traits in reducing pest-related damage. Similarly, the authors of [44,76] discuss the biochemical reactions of specific tea cultivars when subjected to *E. onukii*, demonstrating that some genotypes can synthesize secondary metabolites that deter feeding and promote plant health.

**Figure 6:**
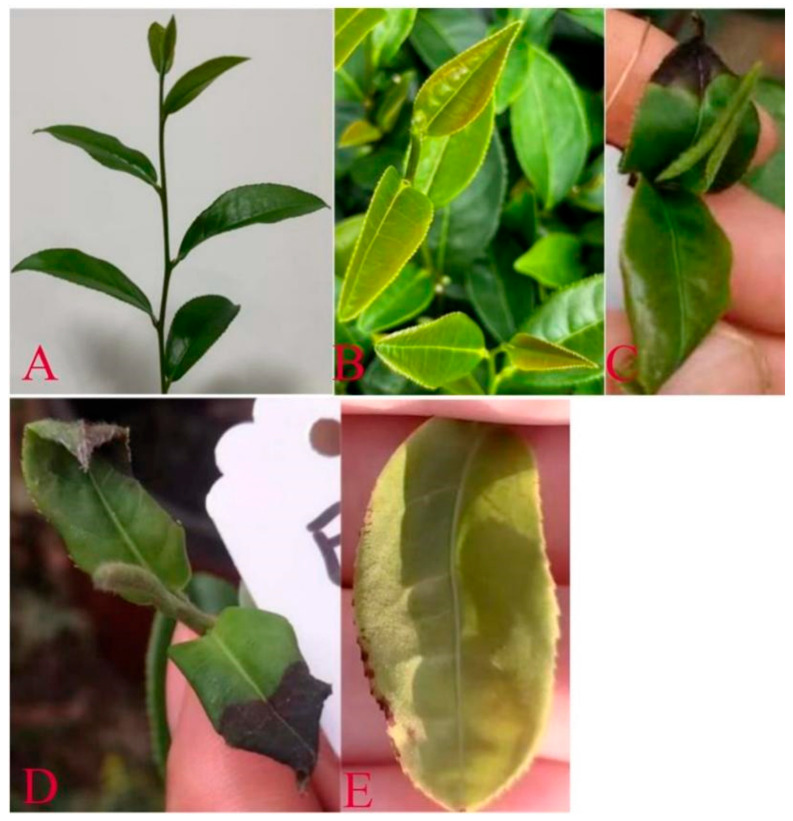
Relationship between leafhopper density and visible leaf damage. (**A**,**B**) Healthy/fresh young tea shoot, (**C**–**E**) the range and types of damage.

Moreover, the continuous feeding by *E. onukii* can lead to visible damage on the tea leaves, manifesting as necrosis (tissue death) and chlorosis (yellowing), as highlighted by [70,81]. Such damage not only reduces tea production but also lowers photosynthetic efficiency and overall plant vigor. According to the authors of [76], the feeding activity of *E. onukii* disrupts the plant’s internal nutrient distribution, which is critical for healthy growth and the proper development of new shoots—both essential for producing high-quality tea (Figure 6).

In addition to causing direct damage, *E. onukii* triggers the production of secondary metabolites in tea plants, such as flavonoids, terpenoids, and volatile organic compounds (VOCs). For example, the authors of [82] revealed that these substances are vital to the plant’s defense mechanisms while also contributing to the unique flavor profiles of teas associated with *E. onukii*. These metabolites are key to the production of “Oriental Beauty” tea, renowned for its honey-like flavor. The authors of [35] provided further insights into how the feeding of *E. onukii* can alter the biochemistry of tea leaves, enhancing desirable sensory qualities in the final tea product. The stress induced by the leafhopper’s feeding is reported to result in complex flavor notes, particularly prized in specialty teas.

The intriguing behavior of *E. onukii* is further highlighted by the role of volatile compounds—aromatic chemicals released by plants that significantly influence pest attraction. As noted by the authors of [6], these volatiles act like a beacon, guiding *E. onukii* to optimal feeding locations. Building on this, the authors of [8] explored how these compounds not only determine where the leafhopper feeds but also influence its mating behavior. Their findings indicate that specific phytochemicals, especially green leaf volatiles (GLVs) and floral fragrances, make certain tea plants particularly appealing to the leafhopper. This preference can drastically affect population dynamics, leading *E. onukii* to congregate in areas abundant with these “fragrant” options.

Interestingly, the authors of [8] also pointed out that environmental conditions such as temperature and humidity can affect the emission efficiency of these volatile compounds. This creates a feedback loop: as environmental factors shift; they can alter not only the health of the host plants but also the behavior and development of *E. onukii* over time. The implications of this research are substantial for pest management; by adjusting volatile profiles through various agricultural practices, farmers may find effective ways to deter *E. onukii* or disrupt its mating behaviors.

In conclusion, the influence of volatile compounds extends far beyond merely attracting *E. onukii*; these aromatic blends also shape feeding habits and reproductive strategies. By understanding these chemical signals, researchers and farmers can develop more effective and environmentally friendly pest management strategies. This knowledge not only aids in managing *E. onukii* in tea plantations but also fosters sustainable farming practices that protect crops while minimizing reliance on chemical interventions.

## 8. Economic Impact of *E. onukii* on the Tea Industry and Farmers

Once regarded as a pest on tea plantations, *E. onukii* has recently been recognized for its potential economic benefits, especially in the production of specialty teas that are highly sought after. This shift in perspective underscores the complex role that *E. onukii* plays in tea production. When managed appropriately, its presence can contribute to the production of unique tea products that command high prices. Enhancing the flavor profile of some tea varieties is one of *E. onukii’s* most important potential uses. The leafhopper’s feeding activity causes the tea plant to go under stress, which causes it to produce secondary metabolites including terpenoids and flavonoids as well as volatile organic compounds (VOCs). Teas with distinct and very desirable flavor characteristics, such as flowery, fruity, and honey-like fragrances, can be produced via these biochemical modifications.

A Case Study on the production of “Oriental Beauty” Tea: The production of this high-end, Taiwanese oolong tea is a well-documented example of this phenomenon. According to research by the authors of [6,8], the development of the tea’s distinct honeyed sweetness and flowery aroma depends heavily on *E. onukii*’s feeding activity. In the market, this tea is highly valued and sells at a substantial premium over ordinary oolong teas [6,8,39]. Moreover, specialty teas have their own niche markets since *E. onukii* populations are purposefully managed to improve tea quality. These products are advertised as premium goods and are highly sought after by tea enthusiasts and consumers prepared to pay more for distinctive and premium teas [9,17]. They are frequently linked to traditional and artisanal manufacturing methods. Due to their unique flavor profiles and artisanal appeal, teas associated with *E. onukii* are priced premium.

Tea manufacturers target particular consumer niches to this differentiation, which opens up chances for larger profit margins. For instance, *E. onukii*-associated teas are sold in Taiwan and Japan highlighting their distinctive flavors and age-old manufacturing techniques in an effort to appeal to both local and foreign consumers [17,53]. Furthermore, the capacity for tea growers to generate high-value teas by means of the controlled existence of *E. onukii* may result in amplified financial gains. Farmer income and stability can be increased by concentrating on producing specialty teas that command higher market prices. In comparison to traditional tea farms, research by the authors of [75] shows that tea farms producing leafhopper-linked teas can yield better revenue per unit area. For small-scale growers who would find it difficult to make ends meet given the low margins associated with mass-produced teas, this improvement in profitability is especially crucial [11,49].

This technique is in line with the expanding global trend toward organic and sustainable farming. Some tea producers are cutting back on or stopping the use of chemical pesticides that would normally target the *E. onukii* as demand for these specialty teas rises. This change encourages environmental sustainability in addition to the manufacturing of premium teas. Tea farms can reduce their environmental impact by mass growing *E. onukii* and using fewer pesticides to maintain controlled populations. This strategy can increase the teas’ marketability and appeal, especially to environmentally conscious consumers. According to the authors of [35], this tactic has helped some areas create more environmentally friendly methods of growing tea.

Furthermore, this integrative method presents an opportunity for the growth of tea-related tourism. Tea farms with a reputation for producing these specialty goods may draw visitors eager to sample and experience the art of making tea. This type of agri-tourism not only helps local communities financially but also fosters the cultural legacy connected to the tea industry. The emergence of tea tourism offers an extra source of income, bolstering regional economies and augmenting the industry’s overall economic influence. In order to generate new chances for economic growth, visitors to these places can take part in guided tours and tea tastings and discover how *E. onukii* is used to make premium teas [9,17,77]. *E. onukii* has the potential to have a significant positive economic influence on the tea sector, especially if its presence is strictly controlled to improve the caliber of tea products. It can change from a common pest to a useful tool for tea growers by helping to generate distinctive flavor profiles, establishing specialized markets, and encouraging sustainable practices. This creative strategy offers new opportunities for sustainable economic growth and development while also boosting farmers’ profits and fostering the expansion of the tea sector overall [22].

## 9. Beneficial Compounds Associated with *E. onukii*

The relationship between *E. onukii* and tea plants (*Camellia sinensis*) offers a fascinating example of how herbivory can transform crop quality (Table 2). While *E. onukii* is traditionally regarded as a pest, its feeding initiates complex biochemical and molecular responses in tea plants that significantly enhance the sensory and functional attributes of the tea. These enhancements occur both in the field and during post-harvest storage, resulting in improved flavor, aroma, and preservation characteristics.

### 9.1. Improvements in the Field

Research by the authors of [8,55] highlights that *E. onukii* feeding stimulates the production of volatile organic compounds (VOCs), including linalool, geraniol, and methyl salicylate. These VOCs not only impart floral, fruity, and minty aromas that define premium teas such as Oriental Beauty but also serve as semiochemicals, attracting natural predators of *E. onukii*. This dual function promotes ecological balance and reduces the need for chemical pesticides, aligning with sustainable agricultural practices [8].

Additionally, herbivory induces the biosynthesis of polyphenols, especially catechins, which contribute to the bitterness and astringency of green tea—key traits valued in high-quality teas [87]. These polyphenols also have potent antioxidant properties, enhancing the health benefits and shelf stability of the final product.

Stress caused by *E. onukii* feeding further enhances the production of theanine, an amino acid that contributes to tea’s umami and sweet flavors. This creates a harmonious balance with the bitterness of catechins, resulting in a nuanced flavor profile [20,85]. Moreover, the herbivory-induced increase in methyl salicylate (MeSA) adds subtle minty and sweet notes to the tea leaves while modulating the plant’s defensive mechanisms [83].

### 9.2. Enhancements During Post-Harvest Storage

The benefits of *E. onukii*-induced changes extend beyond the field, influencing the quality and longevity of tea during storage. VOCs like linalool and geraniol stabilize the tea’s aroma, ensuring that the floral and fruity notes of premium teas remain vibrant over time [41]. Similarly, polyphenols, particularly catechins, offer strong antioxidant properties that prevent oxidative degradation, extending shelf life and maintaining tea quality [83,87].

Methyl salicylate (MeSA) also plays a crucial role in post-harvest storage by stabilizing volatile compounds and preserving the tea’s sensory characteristics [16,83]. Furthermore, theanine continues to enhance the flavor profile during storage, maintaining the sweetness and umami that appeal to consumers [18].

The herbivory-induced increase in secondary metabolites like polyphenols also improves the appearance of tea leaves, imparting a vibrant color highly sought after in premium teas [41]. This visual appeal, combined with enhanced sensory and functional qualities, adds value to the product.

### 9.3. Implications for Tea Production

The dual roles of *E. onukii* as both a pest and a quality enhancer have profound implications for sustainable tea production. In certain regions, controlled herbivory is intentionally encouraged to produce high-value teas like Oriental Beauty, which command premium prices in global markets. This approach not only reduces pesticide usage but also leverages natural plant–insect interactions to improve crop quality [51,56].

Understanding the biochemical pathways activated by *E. onukii* feeding provides valuable insights for tea producers and researchers, enabling the optimization of cultivation practices. By integrating these findings, it is possible to develop new tea varieties with enhanced flavors, aromas, and storage characteristics, further advancing the global tea industry.

## 10. Future Prospects of Mass Rearing *E. onukii*

The evolving perspective on *E. onukii*, shifting from a predominantly perceived pest to one recognized for its beneficial roles in the tea industry, reflects significant advancements in entomological research. Known for its sap-feeding behavior, *E. onukii* poses challenges to tea plants, particularly in regions with extensive tea cultivation, such as China, Japan, Taiwan, Sri Lanka, and India. The insect primarily consumes phloem sap, resulting in various forms of damage, including leaf discoloration, wilting, and stunted growth due to nutrient depletion. This feeding behavior also heightens the plants’ vulnerability to diseases, as the compromised tissues become more susceptible to infections. In cases of substantial infestations, tea yield may decline, presenting economic difficulties for producers.

Despite these initial adverse effects, emerging research has begun to highlight the potential advantages of *E. onukii*. One key benefit is its contribution to maintaining ecological balance within tea plantations. *E. onukii* can play a role in controlling populations of more harmful pest species by acting as prey for natural predators. Moreover, the phytochemical alterations induced by *E. onukii* feeding can enhance the attractiveness of tea plants to beneficial insects, including predatory species that help manage pest populations effectively.

Another potential advantage of *E. onukii* is its capacity to stimulate the plant’s defense mechanisms. The feeding activities of this leafhopper may boost the synthesis of secondary metabolites in tea plants, such as flavonoids and alkaloids, which can deter other herbivores and enhance the plants’ resistance to pests. Such adaptations contribute to the establishment of healthier and more resilient tea plantations that are better equipped to endure various biotic stresses.

Incorporating *E. onukii* into an integrated pest management (IPM) framework presents exciting possibilities for promoting sustainable agricultural practices. Farmers may harness the positive attributes of this insect to reduce reliance on chemical pesticides, ultimately fostering a healthier ecosystem conducive to tea cultivation while simultaneously enhancing biodiversity.

The mass rearing of *E. onukii* could facilitate these benefits, enabling a better understanding of its ecological roles and supporting its integration into IPM strategies. By developing effective mass rearing techniques, researchers and farmers can ensure a stable population of *E. onukii*, potentially optimizing its positive impact on tea plant health and crop yield. Looking ahead, continued exploration of the various roles *E. onukii* can play within tea ecosystems will be essential for improving sustainable agricultural practices and maximizing the benefits for tea producers worldwide.

## 11. Conclusions

The tea green leafhopper, *E. onukii*, has transitioned from being viewed solely as a pest to being recognized for its significant economic benefits in the tea industry. Recent research shows that its feeding behavior enhances the production of desirable secondary metabolites in tea plants, improving the quality and market value of premium teas, such as Taiwan’s “Oriental Beauty”. This shift is driven by consumer demand for unique flavors and artisanal products, fostering lucrative niche markets for teas influenced by *E. onukii*.

The economic benefits extend beyond flavor enhancement, as sustainable agricultural practices are emerging with many farmers reducing or eliminating pesticide use. This aligns with the growing demand for organic and pesticide-free teas, appealing to health-conscious consumers and benefiting local economies, particularly in rural tea-producing areas.

Research indicates that *E. onukii* populations show varying degrees of susceptibility depending on tea cultivars, environmental factors, and climate conditions, which is crucial for developing integrated pest management (IPM) strategies to balance its beneficial and harmful effects. Climatic factors such as humidity, rainfall, and light also significantly affect *E. onukii* populations and tea production.

The evolving understanding of *E. onukii* highlights the need for continued research into its biology, ecology, and the development of sustainable cultivation techniques. This includes breeding resistant tea varieties, promoting beneficial insects, and exploring innovative farming practices that maximize the economic potential of teas associated with *E. onukii*.

In conclusion, *E. onukii* presents a promising opportunity for economic growth in the tea sector. By leveraging its benefits for tea flavor and quality alongside sustainable management practices, farmers can enhance profitability, support local economies, and contribute to sustainable agriculture. The future of tea production, especially in specialty tea regions, depends on the strategic management of *E. onukii*, showcasing how re-evaluating a pest can reveal its potential as a valuable ally in achieving economic sustainability in the industry.

## Figures and Tables

**Table 1:** Developmental parameter (days) of *E. onukii* reared at different temperatures.

Temperature (°C)	Fecundity (Eggs/Female)	Adult Longevity (Days)	Intrinsic Rate of Increase (r)	Population Doubling Time (Days)	Reference
15	30–40	25–30	0.080	8.66	[38]
20	50–60	20–25	0.150	4.62	[39]
25	70–80	15–20	0.200	3.46	[40]
30	90–100	10–15	0.240	2.88	[39]
35	40–50	5–10	0.100	6.93	[41]

**Table 2:** Key beneficial compounds and their impacts.

Beneficial Compound	Mechanism of Production	Role in Enhancing Tea Quality	Citations
Catechins	feeding by *E. onukii* stimulates the tea plant’s phenolic pathways.	adds bitterness and astringency, essential for the characteristic taste of green tea.	[12,75]
Methyl Salicylate (MeSA)	Generated as part of the plant’s signaling response to herbivory.	Introduces minty and sweet notes to tea aroma while also preventing oxidation, enhancing storage quality.	[65,83,84]
Volatile Organic Compounds (VOCs)	Produced as the plant defends itself from stress caused by herbivory.	Enriches tea’s fruity and floral aroma, elevating its sensory appeal.	[10,60]
Theanine	Stress caused by leafhopper feeding boosts biosynthesis.	Amplifies umami flavors and adds sweetness, making tea more appetizing and premium.	[85,86]
Polyphenols	Herbivory stimulates their accumulation in tea leaves.	Strengthens tea’s antioxidant properties, prolonging freshness during storage and boosting its health benefits.	[33,87]

## Data Availability

Genotype data of *M. onukii* are available on Dryad (https://datadryad.org/stash/share/muyHpreBGuAO-Kanv0KS5L6n7i4p-wgJ6KfgZ6qqFBM).
